# Retraction: Advances in Minimally Invasive General Surgery: A Narrative Review of Techniques, Technologies, and Patient Outcomes

**DOI:** 10.7759/cureus.r248

**Published:** 2026-09-18

**Authors:** Ashok Kumar Verma, Banyeswar Pal, Somen Sanfui, Arnab Mondal, Rahima R Malek, Radhey Shyam Singh, G Harsha Vardhan Reddy

**Affiliations:** 1 Department of General Surgery, Santosh Medical College, Ghaziabad, IND; 2 Department of General Surgery, All India Institute of Medical Sciences, Kalyani, Kalyani, IND; 3 Department of General and Minimally Invasive Surgery, Calcutta Medical Research Institute, Kolkata, IND; 4 Department of Gastrointestinal and Hepato-Pancreato-Biliary Surgery, Calcutta Medical Research Institute, Kolkata, IND; 5 Department of Biochemistry, Dr. N.D. Desai Faculty of Medical Science and Research, Dharmsinh Desai University, Nadiad, IND; 6 Department of General Surgery, All India Institute of Medical Sciences, Jodhpur, Jodhpur, IND; 7 Department of Hepato-Pancreato-Biliary Surgery and Liver Transplantation, Institute of Liver and Biliary Sciences, New Delhi, IND

This article has been retracted by the Editor-in-Chief. A post-publication review established that references cited in the article do not support the statements attributed to them, and that the description of the literature search and source verification in the Methods section could not be reconciled with the article's reference list. The article's conclusions are therefore not considered reliable. The authors disagree with the decision to retract.

